# Vitamin D Supplementation and 12-Month Changes in Muscle Function and Physical Performance in Older Adults with Vitamin D Deficiency: A Prospective Observational Cohort Study

**DOI:** 10.3390/nu18152477

**Published:** 2026-07-31

**Authors:** Christina Angelaki, Vasiliki Mamakou, Ioanna A. Anastasiou, Dimitrios Mourkogiannis, Aristotelis Vathis, Michael Angelakis, Georgia Markou, Stavroula-Panagiota Lontou, Konstantinos Makrilakis, Alexander Kokkinos, Nikolaos Tentolouris

**Affiliations:** 1 1st Department of Propaedeutic Internal Medicine, Medical School, National and Kapodistrian University of Athens, Laiko General Hospital, 11527 Athens, Greece; xristina_aggelaki@yahoo.gr (C.A.); anastasiouiwanna@gmail.com (I.A.A.); drvathis@yahoo.com (A.V.); kmakrila@med.uoa.gr (K.M.); akokkinos@med.uoa.gr (A.K.); 2 Department of Internal Medicine, Dromokaiteio Psychiatric Hospital, 12461 Athens, Greece; vmamakou@hotmail.com; 3 Department of Pharmacology, National and Kapodistrian University of Athens, 11527 Athens, Greece; 4 School of Naval Architecture and Marine Engineering, National Technical University of Athens, 15780 Athens, Greece; dmourkogian@gmail.com; 5 Postgraduate Programme “The Interaction Between the Immune System and Kidney Disease: Translational and Clinical Approach”, School of Medicine, Aristotle University, 54124 Thessaloniki, Greece; 6 2nd Department of Vascular Surgery, Medical School, National and Kapodistrian University of Athens, Laiko General Hospital, 11527 Athens, Greece; markou.georgia@gmail.com; 7 1st Department of Internal Medicine, National and Kapodistrian University of Athens, Laiko General Hospital, 11527 Athens, Greece; slondou@gmail.com

**Keywords:** vitamin D supplementation, older adults, muscle function, vitamin D deficiency, sarcopenia, handgrip strength, physical performance, functional decline, musculoskeletal health

## Abstract

Background: Vitamin D deficiency in older adults may impair muscle function and physical performance. This prospective observational cohort study examined the 12-month outcomes of vitamin D supplementation, delivered as part of routine clinical care, in older adults with vitamin D deficiency compared with an insufficiency comparison group. Methods: A total of 194 participants aged ≥60 years were enrolled: 164 with vitamin D deficiency (25(OH)D <20 ng/mL; supplementation group) and 30 with insufficiency (20–29 ng/mL; comparison group). The supplementation group received supplementation targeting 25(OH)D >30 ng/mL; the comparison group received no supplementation. The Handgrip Strength Test (HGS), the Gait Speed Test (GST), the Short Physical Performance Battery Test (SPPB), and the Timed Up and Go Test (TUG) were assessed at baseline and 12 months. Results: Supplementation raised 25(OH)D by 142.9% versus 5.4% in the comparison group (*p* < 0.001). For the primary outcome, the supplementation group showed a 16.2% increase in the HGS versus no change in the comparison group (*p* < 0.001; ANCOVA partial η^2^ = 0.284). For the secondary outcomes, the comparison group was associated with a significant functional decline relative to the supplementation group: the median change on the SPPB in the comparison group was 0.0% (IQR: −8.7, 0.0; between-group *p* < 0.001) and the TUG worsened by +3.2% (between-group *p* < 0.001), while the supplementation group remained stable. The ANCOVA indicated between-group differences on the SPPB (*p* < 0.001) and TUG (*p* = 0.002). Gait speed showed no significant difference. Conclusions: Vitamin D supplementation was associated with more favourable functional outcomes across multiple musculoskeletal domains in vitamin D-deficient older adults. The comparison group’s deterioration on the SPPB and TUG is consistent with an association between correcting deficiency and preserved function.

## 1. Introduction

Vitamin D deficiency poses a significant and well-documented health risk for the elderly population, contributing to a cascade of adverse outcomes that include frailty and sarcopenia [1,2,3,4]. Older adults are vulnerable to developing vitamin D deficiency due to reduced sunshine exposure, decreased skin capacity to synthesise vitamin D, impaired absorption and renal activation, inadequate dietary intake, increased body mass index (BMI), institutionalisation, and presence of chronic diseases [5,6,7,8].

Vitamin D deficiency has been associated with reduced muscle strength, decreased muscle mass, impaired muscle function, diminished bone mineral density, and overall reduced physical performance [9,10,11,12,13,14]. Muscle weakness and loss can significantly increase the risk of falls, frailty, and fractures, ultimately leading to a loss of independence in the elderly [15,16,17].

Vitamin D supplementation has been extensively investigated as a strategy to improve muscle function and maintain physical performance in aging adults [18,19,20]. Vitamin D is believed to affect muscle health via both direct and indirect mechanisms. Indirectly, it plays a crucial role in calcium and phosphate homeostasis [21,22]. Directly, vitamin D modulates skeletal muscle signalling through activation of the vitamin D receptor (VDR) [23,24,25]. Notably, while the VDR concentration decreases with age [26], vitamin D supplementation increases the VDR concentration in skeletal muscles [27,28].

However, the evidence on whether vitamin D supplementation improves muscle function in older adults remains mixed [29,30]. These inconsistent findings likely reflect differences across studies in participant characteristics (e.g., age, comorbidities, and lifestyle), baseline vitamin D status, and supplementation protocols (dose, frequency, and duration). Meta-analyses have indicated that supplementation may benefit older adults with very low baseline 25(OH)D levels (<10 ng/mL) [29,30], but the results are less consistent in the broader community-dwelling elderly population [31]. Notably, some trials have reported a higher rate of falls with large bolus doses of vitamin D [32]. Overall, definitive evidence on both the efficacy of and the optimal dosing strategy for vitamin D supplementation for improving muscle function and reducing falls is still lacking [33].

Given the high prevalence of vitamin D deficiency among older adults [34,35], correcting deficiency remains an important strategy not only to prevent bone disease, but to also potentially preserve muscle function with aging. Accordingly, this prospective observational study evaluated whether vitamin D supplementation, delivered as part of routine clinical care in vitamin D-deficient older adults, was associated with improved muscle function and physical performance, using a comparison group to enable a rigorous assessment of this association.

## 2. Methods

### 2.1. Study Design and Participants

This was a prospective observational cohort study of older adults with vitamin D deficiency or insufficiency managed in routine clinical care. All decisions regarding vitamin D supplementation, including initiation, dosing, and dose adjustment, were made by the treating physicians as part of standard clinical management, independently of and without influence from study participation. The role of the investigators was limited to the prospective observation and systematic collection of data on the outcomes assessed during routine care. Participants were recruited from the outpatient clinics of the Laiko General Hospital, Athens, Greece. The study enrolled individuals meeting the following inclusion criteria: age ≥ 60 years (men and women), vitamin D deficiency defined as serum 25(OH)D levels < 20 ng/mL (<50 nmol/L) [36] and vitamin D insufficiency defined as 25(OH)D levels of 20–29 ng/mL (50–75 nmol/L) [37].

Participants were excluded if they had stage 4 or 5 chronic kidney disease [estimated glomerular filtration rate (eGFR) < 30 mL/min/1.73 m^2^] [38], severe liver disease, neuromuscular disorder, inflammatory bowel disease, history of bariatric surgery, radiation enteritis, myopathies, osteoporosis, or bone fractures or hip replacement/spinal surgery within the past 3 months. Individuals with a recent history of vitamin D supplementation or current treatment for vitamin D deficiency were also excluded. The study was conducted according to the principles of the Declaration of Helsinki and was approved by the ethics committee of our hospital [39]. The purpose of the study was clearly explained to all participants and written informed consent was obtained.

### 2.2. Screening and Recruitment

A total of 240 consecutive individuals aged ≥ 60 years were screened. During the screening process, potential participants completed a comprehensive questionnaire to gather data about demographics (age, sex, ethnicity), lifestyle characteristics (physical activity levels, smoking status, alcohol intake), anthropometric measures, medical history, mini-mental state examination (MMSE) scores [40], consumption of vitamin D-rich foods, and sun exposure habits. Following an overnight fast, blood samples were collected from all eligible participants to measure serum 25(OH)D concentrations and conduct biochemical analyses. The serum 25(OH)D was quantified by an electrochemiluminescence immunoassay (ECLIA) on a Roche Cobas 6000 analyser (Roche Diagnostics International Ltd., Rotkreuz, Switzerland). The same assay and platform were used for all subsequent 25(OH)D measurements throughout the follow-up (Section 2.4). Among the screened individuals, 46 were excluded: 4 did not consent to participate, 36 were taking vitamin D supplements, and 6 had chronic kidney disease.

Thus, 194 individuals were recruited for the study. Supplementary Table S1 presents the questionnaire scores. The chronic disease variable (used as the comorbidity covariate in the fully adjusted models) was derived from the screening questionnaire as a count of each participant’s coexisting, physician-diagnosed chronic diseases, top-coded at a maximum of 3 (“3” denoting three or more). The chronic diseases counted toward this variable are listed in Supplementary Table S3. The compressed interquartile range therefore reflects this ceiling rather than uniform multimorbidity; across the sample, 69, 28, and 97 participants had 1, 2, and ≥3 diseases, respectively, with no between-group difference (*p* = 0.719). Participants were community-dwelling outpatients attending routine clinical care and were not enrolled in any institutional or supervised nutritional support programme.

### 2.3. Study Groups and Clinical Context

A total of 164 participants with serum 25(OH)D levels <20 ng/mL who received supplementation as part of routine care formed the supplementation group, while 30 participants with serum 25(OH)D levels of 20–29 ng/mL who were managed expectantly formed the comparison group. The grouping reflected each participant’s baseline vitamin D status and the clinical care they received, rather than any assignment, allocation, or influence by the investigators. Due to the pragmatic, real-world nature of this observational study and the ethical considerations inherent to a clinical setting, the supplementation group had a mean age of 71.91 years and the comparison group 73.97 years (Table 1), a population for whom vitamin D deficiency carries well-established risks of muscle loss, falls, and fractures. A randomised placebo-controlled design was not implemented; withholding clinically indicated supplementation from older adults with a confirmed deficiency would not have been consistent with established guidelines or the duty of care owed to this vulnerable population. The comparison group comprised older adults with vitamin D insufficiency (20–29 ng/mL) who, at the time of enrolment, did not meet the clinical threshold for mandatory supplementation in this primary care context. This design therefore reflects a real-world comparison between a deficiency group receiving clinically indicated treatment and an insufficiency group managed expectantly over 12 months. The non-randomised grouping and the resulting baseline non-equivalence between groups is acknowledged as a limitation; ANCOVA models with covariate adjustment were constructed to account for these differences, with a baseline value adjustment applied as the default approach and the covariate substitution procedure determined during the analysis (described as post hoc in the Covariate Adjustment (ANCOVA) Section; Table 4a). The supplementation group received vitamin D (cholecalciferol 25,000 IU/week), targeting serum 25(OH)D levels above 30 ng/mL. The comparison group received no vitamin D supplementation. Muscle strength and physical performance were assessed in all participants regardless of group.

### 2.4. Clinical Follow-Up and Routine Dose Management

The follow-up schedule and dose-adjustment steps described below reflect the standard clinical approach used by the treating physicians to manage vitamin D deficiency at our institution; they were followed as part of routine care and were not modified for research purposes. The study recorded these routine visits and measurements but did not dictate them. Throughout the study, the supplementation group participants attended follow-up visits at 3, 6, 9, and 12 months. The comparison group participants attended a single follow-up visit at 12 months, at which the serum 25(OH)D levels, muscle strength, and physical performance were assessed; no intermediate biochemical monitoring or dosage adjustments were applicable given that this group received no supplementation. At the 3-month visit, blood samples were collected from the participants in the supplementation group for a serum 25(OH)D analysis. The serum 25(OH)D concentrations were measured using an electrochemiluminescence immunoassay (ECLIA) on a COBAS analyser (Cobas 6000, Roche Diagnostics International Ltd., CH-6343, Rotkreuz, Switzerland). In the supplementation group, if the 25(OH)D levels were >30 ng/mL, participants received a maintenance dose of vitamin D (cholecalciferol 25,000 IU every 2 weeks); if the levels were ≤30 ng/mL, the dose was increased to cholecalciferol 25,000 IU five times per month. At the 6-month visit, the serum 25(OH)D levels were measured again in the supplementation group. Dosage adjustments followed the same approach as at 3 months: if the 25(OH)D levels were >30 ng/mL, participants received cholecalciferol 25,000 IU every 2 weeks; if levels were ≤30 ng/mL, the dose was increased to cholecalciferol 25,000 IU six times per month. At the 9-month visit, the serum 25(OH)D was measured in the supplementation group, and if levels were ≤30 ng/mL, the participants received cholecalciferol 25,000 IU twice per week. A maintenance regimen of cholecalciferol 25,000 IU every 2 weeks was administered to participants whose 25(OH)D levels were >30 ng/mL. At the final 12-month visit, the serum 25(OH)D levels, muscle strength, and physical performance were assessed in all participants in both the supplementation and comparison groups. Overall, this dose-adjustment approach reflects the treat-to-target strategy used in routine practice to achieve and maintain serum 25(OH)D above 30 ng/mL.

### 2.5. Outcome Measures

Muscle function and physical performance were assessed at baseline and at the 12-month visit.

The primary outcome was the 12-month between-group change in muscle strength, measured by the Handgrip Strength Test (HGS), in accordance with the EWGSOP2 criteria [41]. The HGS was selected as the primary outcome because it is a guideline-endorsed marker of muscle strength and sarcopenia, while also being rapid, low cost, and easy to perform and standardise in real-world clinical settings. The secondary outcomes included functional mobility (Timed Up and Go Test, TUG), gait speed over 4 m (Gait Speed Test, GST), and overall physical performance (Short Physical Performance Battery Test, SPPB). Changes in serum 25(OH)D over 12 months were also evaluated. Instrument selection was aligned with the EWGSOP2 sarcopenia framework [41], which designates the HGS as the primary measure of muscle strength and the SPPB, GST, and TUG as measures of physical performance. Although a muscle mass assessment (Criterion 2) was beyond the scope of this study, this instrument set allowed for clinically interpretable findings within the sarcopenia literature and direct comparability with musculoskeletal ageing research.

#### 2.5.1. Measurement of Muscle Function

Muscle function is underlined by the three concepts of muscle strength, muscle power and muscle endurance [42].

For measuring muscle strength, the Handgrip Strength Test (HGS) was used [43] using a Jamar hydraulic dynamometer following a standardised protocol [43]. The HGS is a reliable measure of overall muscle strength. The best of three trials on each hand was recorded and the result was expressed in kilograms (kg).

#### 2.5.2. Measurement of Physical Performance

For measuring physical performance, the GST, the SPPB and the TUG were assessed [42]. The Gait Speed Test was assessed over a 4 m course. Participants were instructed to walk at their usual pace, and the time required to complete the distance was recorded, allowing for acceleration and deceleration phases. This test serves as an indicator of lower extremity function and overall mobility [44,45]. The SPPB test is an objective measurement instrument of balance, lower extremity strength, and functional capacity in older adults. The test includes three different domains, walking, sit-to-stand and balance, to assess functional mobility [46,47,48]. SPPB testing was performed following established procedures with a well-defined rating scale. The TUG test was measured as the time taken to stand up from a chair, walk 3 m, turn around, and return to a seated position; the TUG test assesses gait, balance, and functional mobility [49,50,51].

### 2.6. Outcome Assessment and Masking

As supplementation was delivered as part of routine clinical care, participants were aware of whether they were receiving vitamin D. To minimise potential bias, all assessments of muscle function and physical performance, as well as the biochemical analyses, were performed by personnel who were blinded to each participant’s group.

### 2.7. Statistical Analysis

All data were entered into a computerised database and analysed using the statistical package for the social sciences (SPSS; IBM Corp., IBM SPSS Statistics for Windows, Version 29.0, Armonk, NY, USA). Depending on the data distribution, either parametric (independent samples *t*-tests) or non-parametric tests (Mann–Whitney U test) were used; a Chi-square test was used for the categorical variables. Within-group changes from baseline to 12 months were analysed using paired *t*-tests or Wilcoxon signed-rank tests, as appropriate. A two-tailed *p* value < 0.05 was considered statistically significant.

Given that five outcome variables were analysed, a Benjamini–Hochberg false discovery rate correction was applied as a secondary sensitivity analysis to control for Type I error inflation from multiple comparisons. All four significant ANCOVA-adjusted outcomes (serum 25(OH)D, HGS, SPPB, and TUG) retained significance after FDR correction (adjusted α thresholds: 0.0100–0.0500); the GST remained non-significant.

#### Covariate Adjustment (ANCOVA)

Given that the two groups were not fully equivalent at baseline, ANCOVAs were used to produce adjusted group comparisons for all five outcomes. Before running each model, the homogeneity of regression slopes was verified via a Group × covariate interaction test (*p* > 0.05 required). The baseline 25(OH)D served as the default covariate for the SPPB and TUG—a statistical rather than mechanistic choice—confirmed to meet the homogeneity requirement for both (*p* = 0.262 and *p* = 0.197, respectively); using each outcome’s own baseline was ruled out as both failed the check (*p* = 0.045 and *p* < 0.001). For the HGS, the baseline HGS replaced the default covariate given the substantial between-group difference at enrolment (median: 24.00 vs. 28.00 kg; *p* = 0.020; R^2^ = 0.894), with homogeneity confirmed (*p* = 0.141). For the GST, the baseline 25(OH)D failed the check (*p* = 0.003), so the baseline GST was used instead (*p* = 0.506). For the serum 25(OH)D itself, the baseline 25(OH)D likewise failed (*p* < 0.001)—expected, given that the groups were defined by their starting vitamin D levels—so the baseline TUG was used instead (*p* = 0.437); this substitution was statistical rather than mechanistic. The adjusted least-squares means with 95% confidence intervals are reported alongside the F-statistics and partial eta-squared (η^2^) as the effect size. The Type II sum of squares was used throughout. All results are presented in Table 4a. The study design and the choice of handgrip strength as the primary outcome were defined at the study’s outset. Each outcome was adjusted for its own baseline value; where the homogeneity of regression slopes assumption was violated, an alternative covariate was substituted. Because these covariate substitutions were determined during the analysis and several (notably the 25(OH)D adjusted for the baseline TUG, and the SPPB and TUG adjusted for the baseline 25(OH)D) are statistical rather than physiologically motivated, they are regarded as post hoc; the corresponding ANCOVA estimates are treated as exploratory, and the covariate selection-independent mixed-effects analysis (Table 4c) serves as the primary robustness check; it yielded concordant conclusions.

As a further secondary sensitivity analysis, each outcome was additionally re-analysed using a fully adjusted ANCOVA model that included, alongside the primary baseline covariate, age, sex, BMI, comorbidity (chronic disease count), physical activity, sun exposure, and dietary vitamin D intake. This assessed the robustness of the baseline-adjusted estimates to potential residual confounding from the measured baseline imbalances. Results of these fully adjusted models, with 95% confidence intervals, are reported in Table 4b.

## 3. Results

**Figure 1:**
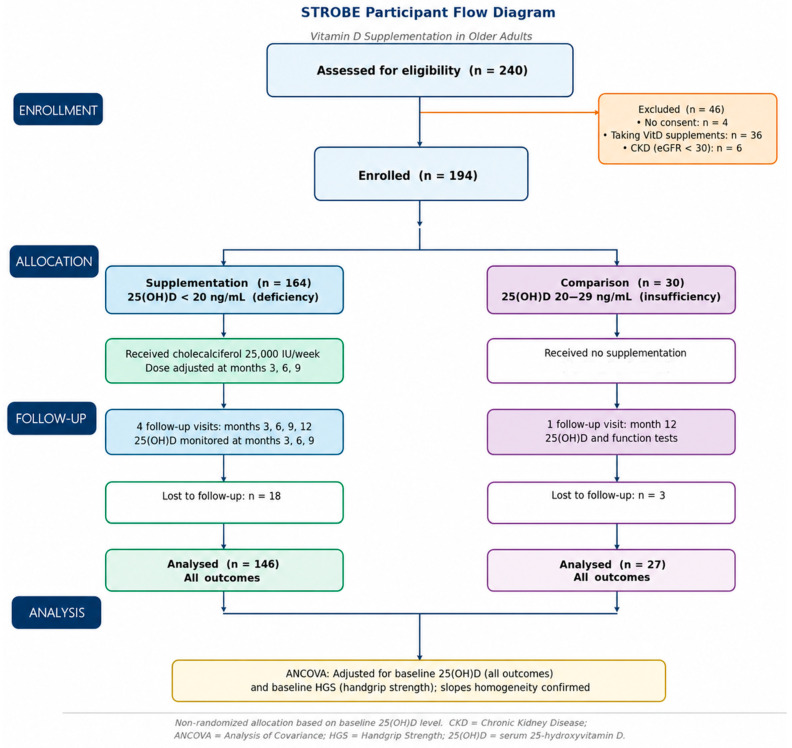
STROBE-compliant participant flow diagram. Of 240 individuals screened, 46 were excluded (4 declined consent; 36 were receiving vitamin D supplementation; 6 had CKD eGFR < 30). The remaining 194 were divided into the supplementation group (n = 164; 25(OH)D < 20 ng/mL) or comparison group (n = 30; 25(OH)D of 20–29 ng/mL) based on their baseline vitamin D status. Grouping was non-randomised. Of 164 supplementation participants, 146 completed the 12-month visit (18 lost to follow-up); of 30 comparison group participants, 27 completed follow-up (3 lost to follow-up). Total analysed: n = 173. ANCOVA analyses adjusted for baseline covariates (Table 4a).

The STROBE-compliant participant flow diagram is presented in Figure 1. The demographic and clinical characteristics of the study participants in the supplementation (n = 164) and the comparison groups (n = 30) are summarised in Table 1. There was no significant difference between the participants taking the vitamin D supplements and the comparison group in terms of age, gender, smoking habits, BMI, health status, multidrug use, MMSE scores, history of falls, exercise habits, sun exposure, and presence of chronic diseases (all *p* > 0.05). The participants in the supplementation group more frequently consumed foods rich in vitamin D in comparison with the comparison group [median value (interquartile range, IQR): 3.00 (2.00, 3.00) vs. 2.00 (2.00, 3.00), respectively, *p* = 0.032].

Table 2 presents a comparison of baseline measurements for the key variables between the supplementation and comparison groups. These variables include the serum 25(OH)D levels and the results of the HGS, GST, SPPB, and TUG tests. The serum 25(OH)D concentrations, HGS, SPPB score, and TUG showed significant baseline differences between groups (*p* < 0.001, *p* = 0.020, *p* < 0.001, and *p* = 0.038, respectively), whereas the GST did not differ significantly at baseline (*p* = 0.326).

**Figure 2:**
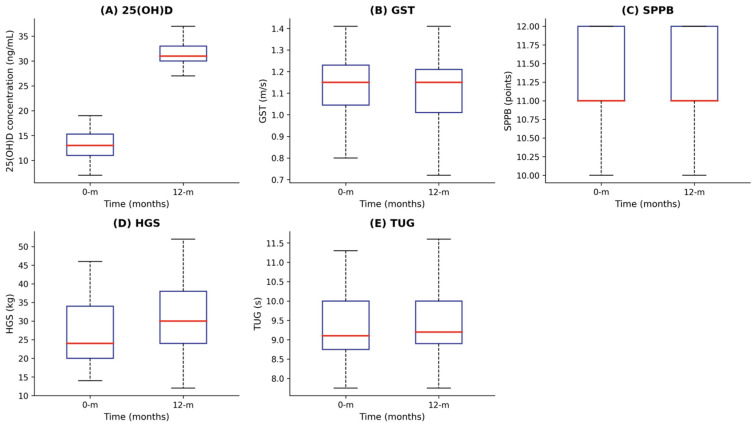
Box plots showing absolute values of serum 25(OH)D (ng/mL), gait speed (GST, m/s), SPPB (points), handgrip strength (HGS, kg), and Timed Up and Go (TUG, s) in the supplementation group at baseline (0-m) and 12 months (12-m). The red central line indicates the median. The left box represents baseline (0 months) and the right box 12 months for each panel.

Absolute serum 25(OH)D concentrations and functional outcome values in the supplementation group at baseline and 12 months are shown in Figure 2.

Table 3 presents the % change from baseline (0) to 12 months for each group separately, alongside between-group comparisons of those changes. During follow-up, serum 25(OH)D levels remained within safe limits in all participants (no values >100 ng/mL), and no supplementation-related toxicity was observed (see Limitations regarding safety monitoring). Supplementary Table S2 presents the serum 25(OH)D concentrations in each group.

**Figure 3:**
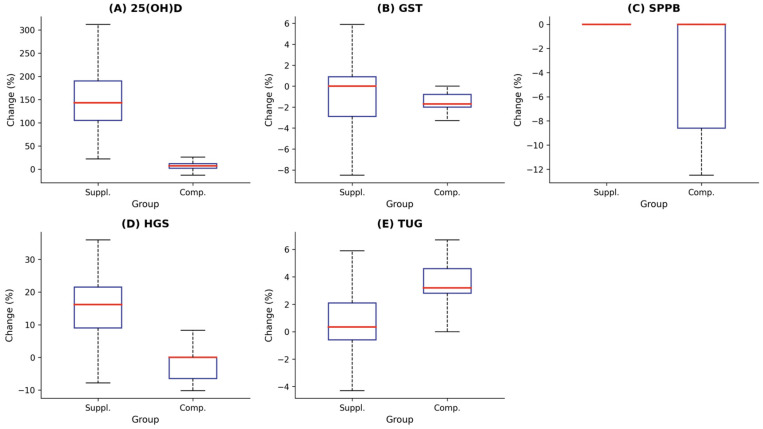
Percentage change from baseline to 12 months, stratified by group (left box: supplementation, n = 146; right box: comparison, n = 27) for 25(OH)D, GST, SPPB, HGS, and TUG. Plots display percentage change (%). Median (red central line), IQR (box), and range (whiskers) are shown. Vitamin D: serum 25(OH)D; HGS: Handgrip Strength; GST: Gait Speed Test; SPPB: Short Physical Performance Battery Test; TUG: Timed Up and Go Test.

Figure 3 displays box plots illustrating the % change in serum 25(OH)D and functional measures between participants in the supplementation and the comparison groups over 12 months.

Covariate-Adjusted Analyses (Table 4a): To address the non-randomised baseline differences in serum 25(OH)D and HGS, ANCOVA analyses were performed. The adjustment for the baseline 25(OH)D showed significant between-group differences in the SPPB (F = 14.273, *p* < 0.001), TUG (F = 10.217, *p* = 0.002), and serum 25(OH)D (F = 80.745, *p* < 0.001; covariate: baseline TUG). The HGS was non-significant when adjusted for the baseline 25(OH)D alone (F = 2.005, *p* = 0.158); however, when adjusted for the baseline HGS—the clinically appropriate covariate given the significant baseline imbalance—the supplementation group showed a significantly greater 12-month HGS score (31.41 vs. 26.71 kg; F = 67.358, *p* < 0.001; partial η^2^ = 0.284). For the GST, the homogeneity of regression slopes assumption was violated for the VitD_0_-adjusted ANCOVA (interaction F = 8.81, *p* = 0.003); using the baseline GST as the covariate instead, no significant difference was found (*p* = 0.386).

The absolute SPPB scores were essentially stable in the supplementation group (mean 11.10 to 11.05; median 11 [IQR 11–12] at both time points) but declined in the comparison group (mean 10.70 to 10.41; median 11 [IQR 10–11]). The score distribution was concentrated in the high-performance band throughout: at baseline, 89.7% of the supplementation group and 88.9% of the comparison group scored 10–12, with the remainder in the 7–9 band (8.9% and 11.1%, respectively) and almost none in the 0–6 band. Reflecting a pronounced ceiling effect, 47.3% of the supplementation group and 11.1% of the comparison group scored the maximum of 12 at baseline, and ≥11 was recorded in 82.9% and 74.1%, respectively, leaving little room for measurable improvement. Applying a ≥1-point change as the threshold for a clinically meaningful difference, most supplementation-group participants remained unchanged (92.5%), with 1.4% improving and 6.2% declining; in the comparison group, none improved, 70.4% remained unchanged, and 29.6% declined by ≥1 point. This higher proportion of clinically meaningful decline in the unsupplemented comparison group, despite comparable baseline distributions, is consistent with a protective association of correcting a deficiency, although the bounded, ceiling-constrained nature of the scale warrants cautious interpretation. The fully adjusted sensitivity analyses and 95% confidence intervals are presented in Table 4b.

The covariate selection rationale and homogeneity test results are reported in the Covariate Adjustment (ANCOVA) Section.

**Figure 4:**
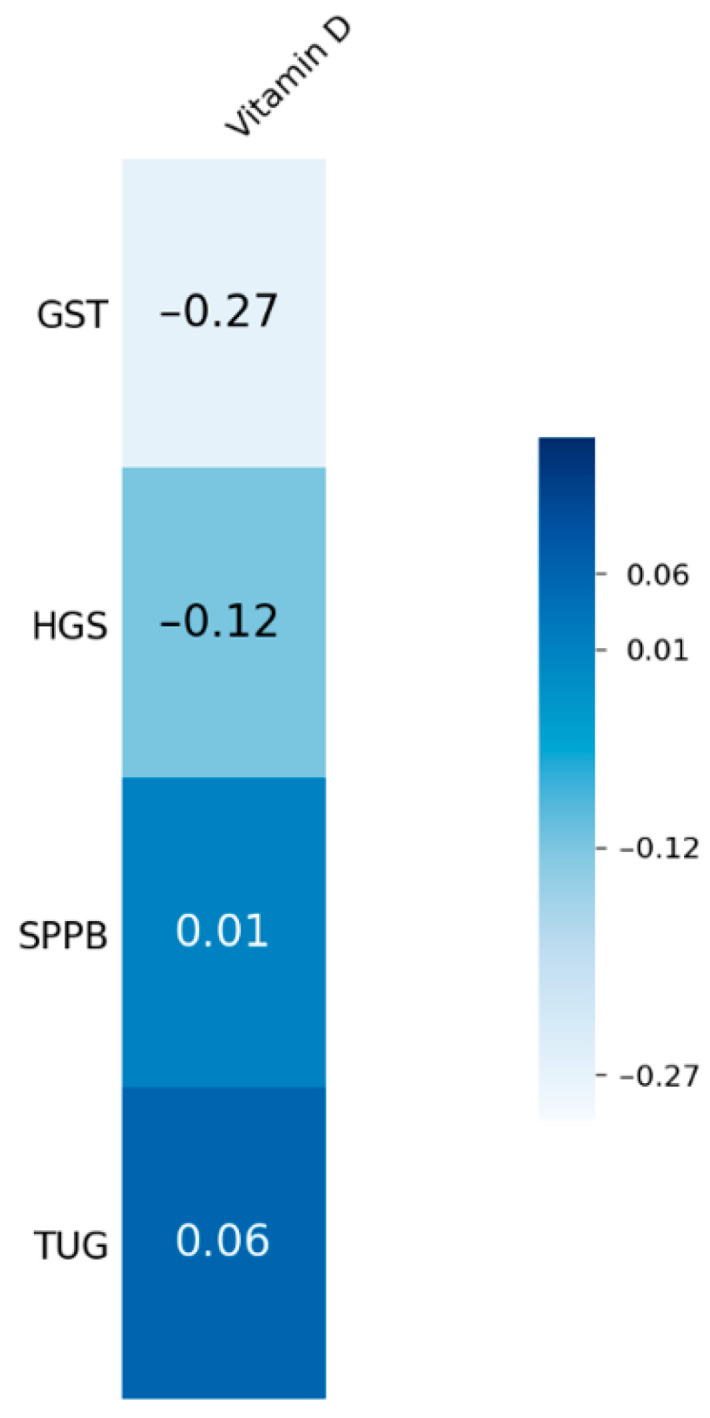
Correlation between serum 25(OH)D % change and functional outcome % changes within the vitamin D supplementation group (n = 146), as visualised by a colour gradient heatmap (Pearson r). Only the GST reached statistical significance (r = −0.27, *p* < 0.001); the HGS (r = −0.12), SPPB (r = +0.01), and TUG (r = +0.06) were not statistically significant. Vitamin D: serum 25(OH)D; HGS: Handgrip Strength; GST: Gait Speed Test; SPPB: Short Physical Performance Battery Test; TUG: Timed Up and Go Test.

The heatmap in Figure 4 reveals a weak and inconsistent relationship between the serum 25(OH)D levels and functional measures within the supplemented group. This is consistent with a categorical rather than linear relationship: the benefit of vitamin D on muscle function appears to depend not on achieving higher concentrations within the sufficient range, but on crossing from deficiency (<20 ng/mL) into sufficiency (>30 ng/mL) as the critical physiological event [52]. The supplementation group precisely accomplished this transition (median 13.00 to 31.20 ng/mL), while the comparison group, remaining within insufficiency throughout (median 24.00 to 26.25 ng/mL), did not—a distinction that maps directly onto the divergent functional trajectories observed between the two groups.

## 4. Discussion

This study investigated the association of vitamin D supplementation with muscle function and physical performance in older adults with vitamin D deficiency. As expected, supplementation significantly raised serum 25(OH)D levels; the supplementation group showed a 142.9% increase versus 5.4% in the comparison group (*p* < 0.001), with an ANCOVA-adjusted means of 31.66 ng/mL (95% CI: 31.17–32.16) versus 25.90 ng/mL (95% CI: 24.74–27.07) (F = 80.745, *p* < 0.001; Table 4a). Among the outcomes assessed, handgrip strength, the primary outcome, showed the most pronounced between-group differences. The supplementation group achieved a median HGS increase of 16.2% (*p* < 0.001; Table 3), supported by the ANCOVA-adjusted means of 31.41 kg versus 26.71 kg (95% CI: 30.97–31.85 vs. 25.67–27.75; F = 67.358, *p* < 0.001; partial η^2^ = 0.284; Table 4a), representing a clinically meaningful and statistically robust association with upper limb muscle strength. The association was equally evident in the secondary functional outcomes, including lower limb function and functional mobility (SPPB and TUG). Notably, the comparison group also started with a significantly lower baseline SPPB distribution. Similarly, the ANCOVA indicated a significant between-group difference in the SPPB (adjusted means: 11.20 vs. 9.60; F = 14.273, *p* < 0.001; Table 4a). Notably, both groups began with high baseline SPPB scores (median 11 out of a possible 12), reflecting a functionally robust cohort near the upper end of the scale; this ceiling effect constrained the capacity to detect further improvement, so the observed SPPB result is best interpreted as stability of function in the supplementation group relative to the decline in the comparison group, rather than as a large gain attributable to supplementation. The TUG time remained stable in the supplementation group (+0.3%) while deteriorating significantly in the comparison group (+3.2% worsening; between-group *p* < 0.001; Table 3), and this pattern persisted after covariate adjustment (adjusted means: supplementation 9.33 s vs. comparison group 11.07 s; F = 10.217, *p* = 0.002; Table 4a). The convergence of Table 3 change score analyses and Table 4a ANCOVA-adjusted estimates across all four outcomes provides mutually reinforcing evidence of a pattern consistent with a protective association of vitamin D supplementation with musculoskeletal function; in the fully adjusted sensitivity analyses (Table 4b), these effects were retained for the 25(OH)D, HGS, and SPPB, while the TUG effect was borderline (*p* = 0.050) and should be interpreted cautiously. Gait speed (GST), a secondary outcome, was the only measure showing no significant difference in either the unadjusted or adjusted analyses. This may reflect a ceiling effect, as the baseline gait speeds of 1.13–1.19 m/s in both groups are near the normal range for community-dwelling older adults, leaving limited room for further improvement or detectable decline within the 12-month timeframe. Moreover, the observed between-group gait speed difference (~0.01 m/s) is well below the reported minimal detectable change for usual gait speed in older adults (0.18 m/s) [53], indicating that any true difference, if present, would fall below the threshold of reliable measurement. Taken together, these findings are consistent with a potential protective association of correcting vitamin D deficiency in older adults, with significant associations with the primary outcome (handgrip strength) and consistent advantages across the secondary functional outcomes (SPPB and TUG). The pattern of functional decline across these secondary outcomes in the unsupplemented comparison group, which was not observed in the supplementation group, may reflect the potential consequences of untreated deficiency on mobility and independence in this population. Given that impaired physical performance is a key predictor of falls, hospitalisation, and loss of independence in older adults, these results are consistent with a potential role for vitamin D supplementation within a comprehensive approach to musculoskeletal health.

To aid clinical interpretation, the observed changes were benchmarked against established thresholds. The adjusted between-group HGS difference of ~4.7 kg approaches the minimal clinically important difference for grip strength (5.0–6.5 kg) [54,55] and exceeds its reported minimal detectable change (~2.9 kg in older adults) [53]. For the TUG, the comparison group approached the ≥12 s threshold associated with increased fall risk (adjusted 11.07 s) [56], whereas the supplementation group remained well below it (9.33 s), although these threshold comparisons should be interpreted with appropriate caution given the observational design and the modest comparison group size.

Several studies have underscored the importance of considering additional factors, such as exercise and nutrition, in conjunction with vitamin D supplementation [57,58]. Antoniak et al. [57] proposed that combined resistance exercise training and vitamin D supplementation might be necessary to elicit meaningful improvements in musculoskeletal health and function.

The magnitude of the handgrip strength association observed here is best interpreted in light of participants’ baseline vitamin D status. Beaudart et al. [30] reported that although the overall effect of supplementation on muscle strength was modest (SMD 0.17), the benefit was significantly greater in individuals with a baseline 25(OH)D below 30 nmol/L (<12 ng/mL) than in those above this level (*p* = 0.02). Our supplementation group began near this threshold at a median of 13.0 ng/mL and, through a treat-to-target approach, reached approximately 31 ng/mL, within the ~20–30 ng/mL (50–75 nmol/L) range identified as optimal for lower extremity function [59]. Consistent with this, the meta-analytic evidence in older adults with sarcopenia indicates that oral vitamin D3 may improve handgrip strength [60].The larger association seen here may thus be consistent with the literature, in which a functional benefit appears concentrated among those with a deficiency who achieve sufficiency rather than among those who are already insufficient, precisely the trajectory of our participants. This interpretation should nonetheless be tempered by the non-randomised design and the modest comparison group size. The observation that a functional benefit appeared to track with a transition from deficiency into sufficiency, rather than incremental gains within the sufficient range, is consistent with a threshold effect; however, given the weak and largely non-significant within-group correlations between the 25(OH)D change and functional outcomes (significant only for the GST), we regard this as a hypothesis-generating observation rather than an established mechanism, warranting confirmation through dedicated future studies.

Finally, individual variability in the response to supplementation may be partly determined at the receptor level. Emerging evidence indicates that vitamin D receptor (VDR) polymorphisms can modulate inflammatory, haematologic, and patient-reported outcomes independently of circulating 25(OH)D: VDR rs2228570 (FokI) is associated with distinct inflammatory profiles and health-related quality of life, and VDR rs7975232 (ApaI) with *C*-reactive protein, neutrophil counts, and SF-36 outcomes in orthopaedic populations [61,62]. Receptor-level variability may thus contribute to the heterogeneous functional responses reported across the literature and represents a promising direction for future research.

It is important to consider these factors when interpreting the results and to explore them in future research to better understand the complex relationship between vitamin D supplementation and physical function in older adults.

Several limitations warrant consideration. This was a non-randomised observational study; the grouping was based on baseline vitamin D status rather than randomisation, creating a fundamental non-equivalence between the groups—the supplementation group had a vitamin D deficiency (<20 ng/mL), while the comparison group had an insufficiency (20–29 ng/mL). The comparison group additionally had a significantly higher baseline HGS (*p* = 0.020), lower baseline SPPB score (*p* < 0.001), and higher baseline TUG (*p* = 0.038). Although the supplementation group reported significantly greater consumption of vitamin D-rich foods (*p* = 0.032), this imbalance is unlikely to have materially influenced the outcomes: dietary vitamin D contributes a relatively small fraction of total vitamin D status compared to supplemental cholecalciferol at 25,000 IU/week, and the adjustment for the baseline 25(OH)D in the ANCOVA models implicitly accounted for the cumulative effect of pre-enrolment dietary intake on vitamin D status. No formal a priori sample size calculation was performed, and the small comparison group (n = 30) limited the unadjusted statistical power. The ANCOVA analyses adjusted for baseline non-equivalence and, together with the post hoc mixed-effects sensitivity analysis (Table 4c), strengthened the robustness of findings, which persisted in the fully adjusted sensitivity analyses (Table 4b) for the 25(OH)D, HGS, and SPPB, while the TUG was borderline (*p* = 0.050); nevertheless, covariate adjustment does not substitute for randomisation, and residual confounding cannot be fully excluded.

The dose-adjustment approach across 12 months, aiming to achieve and maintain serum 25(OH)D concentrations above 30 ng/mL in the deficient participants, means that our findings pertain to a treat-to-target supplementation strategy rather than to a single fixed-dose regimen, and may not be generalisable beyond vitamin D-deficient community-dwelling older adults. Of 194 enrolled participants, 21 (10.8%) were lost to follow-up (18 supplementation, three comparison), yielding a final analysed sample of n = 173. This modest attrition is unlikely to have introduced material bias, as the baseline characteristics did not differ significantly between those who completed follow-up and those lost to follow-up.

As a prospective observational study embedded in routine clinical care, the data collection focused on the pre-specified functional outcomes, baseline covariates, and serial serum 25(OH)D concentrations. Serial calcium, phosphorus, and parathyroid hormone measurements were not systematically captured, as supplementation was managed by treating physicians under standard clinical practice rather than a research-defined safety protocol. Nonetheless, several factors support the regimen’s safety: dosing was standard and physician supervised, the serial 25(OH)D remained within safe limits in all participants (no value exceeded 100 ng/mL), and no adverse events were reported during follow-up.

Regarding adherence, although individual dose-level records were not maintained, the substantial and sustained rise in serum 25(OH)D in the supplementation group (median 13.0 to 31.2 ng/mL) provides an objective biomarker indicating effective uptake. We acknowledge that the absence of systematic mineral homeostasis data and formal adherence logs is a limitation, and dedicated biochemical safety monitoring and adherence tracking are warranted in future studies.

This study has several notable strengths. The 12-month follow-up duration exceeds that of most comparable vitamin D supplementation studies in older adults, providing a clinically meaningful timeframe for detecting functional changes. Muscle function and physical performance were assessed using a comprehensive, validated battery of four instruments (HGS, TUG, GST, and SPPB) aligned with the EWGSOP2 sarcopenia diagnostic criteria, which also provided the clinically grounded hierarchy designating the HGS as the primary outcome and the TUG, GST, and SPPB as secondary outcomes. The assessors performing all functional and biochemical measurements were blinded to the participants’ grouping, minimising measurement bias despite the non-blinded nature of supplementation. The grouping was determined by an objective biological criterion—baseline serum 25(OH)D concentration—rather than clinician preference or patient choice, limiting selection bias at the point of grouping. The transparent application of ANCOVA with homogeneity of slopes verification for all models, combined with a Benjamini–Hochberg false discovery rate correction for multiple comparisons, reflects a level of statistical rigour uncommon in pragmatic studies of this type. Finally, the goal-directed, treat-to-target approach used in routine care aiming to achieve and maintain serum 25(OH)D above 30 ng/mL mirrors real-world clinical practice, enhancing the external validity and direct translatability of findings to primary care settings. The serial serum 25(OH)D monitoring at 3, 6, and 9 months indicated that the participants achieved and maintained target concentrations, and verified that levels remained within safe limits (no values >100 ng/mL) throughout the 12-month follow-up.

## 5. Conclusions

This study shows that vitamin D supplementation raises serum 25(OH)D levels (142.9% vs. 5.4% in the comparison group; *p* < 0.001) and is associated with significantly better outcomes across multiple domains of musculoskeletal function in older adults with a deficiency. The supplementation group showed higher handgrip strength (+16.2% vs. 0%; *p* < 0.001), overall physical performance (SPPB: stable median scores in the supplementation group, whereas the comparison group showed a downward shift in distribution [median 0.0%; IQR: −8.7 to 0.0]), and functional mobility (TUG: median +0.3% vs. +3.2% worsening in the comparison group; *p* < 0.001), while the comparison group deteriorated significantly across all three domains. The ANCOVA-adjusted analyses support these between-group differences, with a particularly large effect on handgrip strength (partial η^2^ = 0.284). Gait speed was the only outcome showing no significant change in either group. The consistent pattern of functional decline in the unsupplemented comparison group underscores the potential protective association of correcting vitamin D deficiency with less decline in mobility and independence in older adults. Future interventions should consider a multi-faceted approach, incorporating exercise and nutritional guidance to maximise musculoskeletal health benefits in this population. Long-term studies are essential to fully elucidate the role of vitamin D supplementation on mobility, fall risk, and overall quality of life in older adults.

## Figures and Tables

**Table 1:** Baseline demographic and clinical characteristics.

Groups	Supplementation n = 164	Comparison n = 30	*p* Value
Age (years)	71.91 ± 6.90	73.97 ± 6.30	0.128 ^#^
Gender (males), n (%)	80 (48.8)	12 (40)	0.430 ^ǂ^
Smoking (yes), n (%)	52 (31.7)	10 (33.3)	0.835 ^ǂ^
BMI (kg/m^2^)	27.75 ± 3.5	27.10 ± 3.56	0.356 ^#^
Health status score	2.00 (2.00, 3.00)	3.00 (2.00, 3.00)	0.668 ^†^
Polypharmacy, yes/no	0 (0, 1.00)	1.00 (0, 1.00)	0.423 ^†^
MMSE score	29.00 (28.00, 30.00)	29.00 (27.50, 30.00)	0.180 ^†^
Falls, yes/no	0 (0, 1.00)	0 (0, 1.00)	0.939 ^†^
Exercise score	2.00 (1.0, 3.00)	2.00 (1.00, 3.00)	0.891 ^†^
Consumption of foods rich in vitamin D score	3.00 (2.00, 3.00)	2.00 (2.00, 3.00)	0.032 ^†^
Sun exposure score	2.00 (2.00, 3.00)	2.25 (2.00, 3.00)	0.483 ^†^
Chronic diseases, n	3.00 (3.00, 3.00)	3.00 (2.00, 3.00)	0.719 ^†^

Data are means ± SD (standard deviation), n (%), or median value (IQR). ^#^ *p* values for comparisons between groups by independent samples *t*-test. ^ǂ^ *p* values for comparisons between groups by chi-squared test. ^†^ *p* values for comparisons between groups by Mann–Whitney U test.

**Table 2:** Baseline comparison of muscle function and physical performance metrics between two groups.

	Supplementation (n = 164)	Comparison (n = 30)	*p* Value
25(OH)D, ng/mL	13.00 (11.00, 15.52)	24.00 (22.25, 26.75)	<0.001
HGS, kg	24.00 (20.00, 34.00)	28.00 (24.00, 38.00)	0.020
GST, m/s	1.15 (1.05, 1.23)	1.19 (1.14, 1.22)	0.326
SPPB, score	11.00 (11.00, 12.00)	11.00 (10.00, 11.00)	<0.001
TUG, sec	9.10 (8.80, 10.00)	9.50 (9.00, 10.50)	0.038

HGS: Handgrip Strength; GST: Gait Speed Test; SPPB: Short Physical Performance Battery Test; TUG: Timed Up and Go Test. Data are median value (IQR). *p* values: Mann–Whitney U test for all variables (non-parametric; normality assessed by Shapiro–Wilk).

**Table 3:** Percentage changes in serum 25(OH)D and functional measures between groups from baseline to 12 months.

Percentage (%) Change	Supplementation (n = 146)	Comparison (n = 27)	*p* Value
dD/D0 (%)	142.9 (106.7, 190.7)	5.4 (0.0, 14.8)	<0.001
dHGS/HGS0 (%)	16.2 (9.2, 21.4)	0.0 (−6.5, 0.0)	<0.001
dGST/GST0 (%)	0.0 (−2.9, 0.9)	−1.6 (−1.7, −0.8)	0.022
dSPPB/SPPB0 (%)	0.0 (0.0, 0.0)	0.0 (−8.7, 0.0)	<0.001
dTUG/TUG0 (%)	0.3 (−0.5, 2.1)	3.2 (2.9, 4.9)	<0.001

d: difference; D: 25(OH)D; HGS: Handgrip Strength; GST: Gait Speed Test; SPPB: Short Physical Performance Battery Test; TUG: Timed Up and Go Test. Data are median value (IQR). *p* values for between-group comparisons by Mann–Whitney U test.

**Table 4:** (**a**) ANCOVA-adjusted between-group comparisons at 12 months. (**b**) Sensitivity analyses: fully adjusted ANCOVA, 95% confidence intervals, and minimum detectable effect. (**c**) Post hoc mixed-effects sensitivity analysis: Absolute change from baseline to 12 months (within group) and between-group differential change (group × time interaction), with 95% confidence intervals.

(**a**)						
**Outcome**	**Covariate**	**Adj. Mean—Supplementation (n = 146) [95% CI]**	**Adj. Mean—Comparison (n = 27) [95% CI]**	**F**	***p*** **Value**	**Partial η^2^**
Serum 25(OH)D (ng/mL)	Baseline TUG ‡‡	31.66 (31.17–32.16)	25.90 (24.74–27.07)	80.745	<0.001	0.322
HGS—Handgrip Strength (kg) †	Baseline HGS	31.41 (30.97–31.85)	26.71 (25.67–27.75)	67.358	<0.001	0.284
SPPB Score §	Baseline 25(OH)D	11.20 (10.97–11.42)	9.60 (8.87–10.33)	14.273	<0.001	0.077
TUG—Timed Up and Go (sec) §	Baseline 25(OH)D	9.33 (9.04–9.62)	11.07 (10.14–12.01)	10.217	0.002	0.057
GST—Gait Speed Test (m/s) ‡	Baseline GST	1.12 (1.11–1.13)	1.11 (1.10–1.13)	0.757	0.386	0.004
(**b**)						
**Outcome**	**n**	**Baseline-Adj. Effect (95% CI)**	***p***	**Fully Adj. Effect (95% CI)**	***p***	**Conclusion**
25(OH)D (ng/mL)	173	+5.76 [4.50, 7.03]	<0.001	+5.83 [4.53, 7.13]	<0.001	Robust
HGS (kg)	173	+4.70 [3.57, 5.83]	<0.001	+4.58 [3.34, 5.82]	<0.001	Robust
SPPB (points)	173	+1.60 [0.77, 2.44]	<0.001	+1.10 [0.30, 1.90]	0.007	Robust
TUG (s)	173	−1.74 [−2.82, −0.67]	0.002	−0.97 [−1.94, 0.00]	0.050	Robust (borderline)
GST (m/s)	173	+0.007 [−0.009, 0.024]	0.386	+0.010 [−0.007, 0.027]	0.244	Non-significant
(**c**)						
**Outcome**	**Δ Supplementation (95% CI)**		**Δ Comparison (95% CI)**	**Between-Group Δ (95% CI)**		***p***
25(OH)D (ng/mL)	+18.49 (17.80, 19.19)		+1.54 (0.57, 2.51)	+16.96 (15.78, 18.13)		<0.001
HGS (kg)	+4.14 (3.67, 4.61)		−0.44 (−0.95, 0.06)	+4.58 (3.90, 5.26)		<0.001
SPPB (points)	−0.05 (−0.10, 0.01)		−0.30 (−0.48, −0.11)	+0.25 (0.06, 0.44)		0.001
TUG (seconds)	+0.03 (−0.03, 0.10)		+0.31 (0.17, 0.44)	−0.28 (−0.42, −0.13)		0.001
GST (m/s)	−0.01 (−0.02, −0.01)		−0.02 (−0.02, −0.01)	+0.01 (−0.00, 0.01)		0.438

† For HGS, baseline HGS was selected as covariate to address significant baseline imbalance (median: 24.00 vs. 28.00 kg; *p* = 0.020). Homogeneity confirmed: Group × HGS_0_ interaction *p* = 0.141. ‡ ANCOVA with baseline 25(OH)D violated homogeneity of regression slopes assumption for GST (Group × VitD_0_ interaction: F = 8.81, *p* = 0.003). Baseline GST was used as alternative covariate; homogeneity confirmed (*p* = 0.506). ‡‡ For serum 25(OH)D, homogeneity of regression slopes was violated when using baseline 25(OH)D as covariate (Group × VitD_0_ interaction: *p* < 0.001). Baseline TUG satisfied the assumption (*p* = 0.437) and was used instead. § Homogeneity of regression slopes confirmed for all remaining VitD_0_-adjusted models: SPPB (*p* = 0.262), TUG (*p* = 0.197). Values are adjusted least-squares means (95% CI); Type II SS. Partial η^2^ benchmarks: small = 0.01, medium = 0.06, large = 0.14. Effect = adjusted between-group difference (supplementation vs. comparison) in native units (25(OH)D, ng/mL; HGS, kg; SPPB, points; TUG, s; GST, m/s). Baseline-adjusted models replicate Table 4a; fully adjusted models add age, sex, BMI, comorbidity (chronic disease count), physical activity, sun exposure, and dietary vitamin D. After full adjustment, all effects that were significant in the primary analysis remained significant (25(OH)D, HGS, SPPB), while TUG was borderline (*p* = 0.050) and should be interpreted cautiously, with effect estimates essentially unchanged, indicating that the findings are independent of baseline covariate distribution. Gait speed (GST) remained non-significant. Minimum detectable effect (GST): With n = 146 in the supplementation group and n = 27 in the comparison group (SD ≈ 0.16 m/s), the study has 80% power (α = 0.05, two-sided) to detect ~0.10 m/s (Cohen’s d ≈ 0.59), so this non-significant result is unlikely to be attributable to insufficient power. Note: Linear mixed-effects models (outcome ~ group × time; participant-level random intercept). This covariate selection-independent analysis is reported as post hoc sensitivity check; conclusions are concordant with ANCOVA-adjusted results in Table 4a,b.

## Data Availability

The raw data supporting the conclusions of this article will be made available by the authors on request.

## Supplementary Materials

The following supporting information can be downloaded at: https://www.mdpi.com/article/10.3390/nu18152477/s1, Table S1: A clear overview of factors related to cognitive health, chronic diseases, medication use, health status, physical activity, sun exposure, dietary habits, and falls; Table S2: Serum 25(OH)D concentrations in each group; Table S3: Chronic conditions counted toward the comorbidity (chronic-disease) variable.

## Abbreviations

25(OH)D	25-hydroxyvitamin D
ANCOVA	Analysis of Covariance
BMI	Body Mass Index
ECLIA	Electrochemiluminescence Immunoassay
eGFR	Estimated Glomerular Filtration Rate
GST	Gait Speed Test
HGS	Handgrip Strength Test
IQR	Interquartile Range
MMSE	Mini-Mental State Examination
SD	Standard Deviation
SPPB	Short Physical Performance Battery
TUG	Timed Up and Go Test
VDR	Vitamin D Receptor
