# Supplementary material for: Vitamin D Supplementation and 12-Month Changes in Muscle Function and Physical Performance in Older Adults with Vitamin D Deficiency: A Prospective Observational Cohort Study
Source: Nutrients. 2026 Jul 31;18(15):2477. doi: 10.3390/nu18152477 (PMC13468331; doi:10.3390/nu18152477)
Supplement: Supplementary file 1 [file nutrients-18-02477-s001.zip › nutrients-4432266-supplementary.pdf]

Supplementary Table S1: a clear overview of factors related to cognitive health, chronic diseases, medication use, health status, physical activity, sun exposure, dietary habits, and falls.

Parameters	Score
MMSE score	Mild cognitive impairment (21-24)
	Normal (25-30)
Chronic diseases (comorbidity)	1 chronic disease (1)
	2 chronic diseases (2)
	3 or more chronic diseases (3)
Polypharmacy (medication use)	5 or more medications no (0), yes (1)
Health status	Moderate (1)
	Good (2)
	Very Good (3)
Physical activity	Mild (1)
	Moderate (2)
	Intense (3)
Sun exposure for 10-15 minutes	0-2 times per week (1)
	3-6 times per week (2)
	Daily (3)
Dietary intake of vitamin D-rich foods	0-2 times per week (1)
	3-6 times per week (2)
	Daily (3)
Falls in the last year	No (0)
	Yes (1)

Supplementary Table S2: serum 25(OH)D concentrations in each group.

Supplementation	baseline	3months	6months	9months	12months
25(OH)D, ng/mL	13.00 (11.00, 15.52)	32.10 (27.50, 34.10)	33.80 (31.20, 35.40)	32.95 (30.80, 34.80)	31.20 (30.20, 35.10)

Comparison	baseline	12months
25(OH)D, ng/mL	24.00 (22.25, 26.75)	26.25 (22.30, 27.80)

Data are median value (IQR).

Supplementary Table S3: Chronic conditions counted toward the comorbidity (chronic-disease) variable.

Chronic Diseases
Hypertension
Coronary artery disease
Obesity
Diabetes
Arthritis
Chronic respiratory disease (COPD/asthma)
Cancer (excluding skin cancer)
Thyroid diseases
Anxiety disorders
Depression
Chronic skin conditions
